# Oct4 Regulates the Transition of Cancer Stem-Like Cells to Tumor Endothelial-Like Cells in Human Liver Cancer

**DOI:** 10.3389/fcell.2020.563316

**Published:** 2020-09-30

**Authors:** Hong-Lin Liu, Hong-ting Tang, Han-lin Yang, Ting-Ting Deng, Ya-Ping Xu, Shi-Qing Xu, Liang Peng, Zai Wang, Qing Fang, Xiao-Yan Kuang, Qin-Shan Li

**Affiliations:** ^1^Institute of Clinical Medical Sciences, China-Japan Friendship Hospital, Beijing, China; ^2^Institute of Clinical Pathology, Zunyi Medical College, Zunyi, China; ^3^Department of Obstetrics and Gynecology, Prenatal Diagnosis Center, Affiliated Hospital of Guizhou Medical University, Guiyang, China; ^4^Department of Clinical Biochemistry, School of Clinical Laboratory Science, Guizhou Medical University, Guiyang, China

**Keywords:** Oct4, vasculogenesis, cancer stem-like cells, endothelial trans-differentiation, transition

## Abstract

Octamer-binding transcription factor 4 (Oct4) has been recently implicated as a proangiogenic regulator in several induced pluripotent stem cells (iPSCs), however, its role in cancer stem-like cells (CSCs) remain unclear. We report here that Oct4 participates in tumor vasculogenesis in liver CSCs (LCSCs). We identify that LCSCs possess the potential of endothelial *trans*-differentiation under endothelial induction, present endothelial specific markers and their functions *in vitro*, and participate in neovasculogenesis *in vivo*. The knockdown of the Oct4A by short hairpin RNA (shRNA) in LCSCs represses endothelial *trans*-differentiation potential, but induces endothelial lineage-restricted differentiation, the latter is positively regulated by Oct4B1. Furthermore, Oct4 regulates vasculogenesis in LCSCs may be via the AKT-NF-κB-p65 signaling pathway. This work reveals Oct4, which is a crucial regulator, plays a critical role in tumor endothelial-like cells transition of LCSCs through Oct4A and Oct4B1 by different ways. The simultaneous inhibition of both the isoforms of Oct4 is hence expected to help regress neovascularization derived from CSCs. Our findings may provide insights to the possible new mechanisms of tumor vasculogenesis for primary liver cancer.

## Introduction

Primary liver cancer is the third leading cause of mortality due to cancer across the world (Bray et al., 2018). High vascularization is a histological feature of liver cancer, which in turn is associated with increased vascular invasion and poor prognosis (Imai et al., 2018). Therefore, antiangiogenic strategies of inhibiting tumor endothelial cells (TECs) are considered critical for optimizing the therapeutic effect in liver cancers. It has been proposed earlier that TECs are mainly derived from sprouting of the pre-existing host vessels via angiogenesis, which were dependent on vascular endothelial growth factor (VEGF) and its receptors (VEGFRs) (Jain and Carmeliet, 2012). However, the current therapeutic effects of anti-VEGF/VEGFRs agents are transient and, eventually, bring about drug resistance in a substantial patient population, including that of liver cancer patients (Sampat and O’Neil, 2013; Haibe et al., 2020). It is therefore urgent to elucidate the novel molecular mechanisms of tumor neovascularization and the origin of TECs toward making a breakthrough against the limitations of anti-angiogenic therapy. Some recent studies have proposed that CSCs serve as vascular progenitors, which is of probable importance as a source of TECs, and participate in vasculogenesis (Ricci-Vitiani et al., 2010; Wang R. et al., 2010; Markowska et al., 2017; Baisiwala et al., 2019), these reports may explain the resistance of CSCs to anti-angiogenic agents. Until date, whether LCSCs possess the potential to transit into vascular endothelial cells remains unclear. Thus, the exploration of the endothelial transition potential of LCSCs and its molecular mechanism is expected to open new avenues toward the development of anti-vasculogenic therapy against liver cancer.

Oct4 (also known as POU5F1) is a member of the POU family (Sterneckert et al., 2012). The latest research findings indicate that human Oct4 generates at least three transcript variants via alternative splicing. The corresponding protein isoforms (Oct4A, Oct4B, and Oct4B1) exhibited distinct subcellular distribution, specifically, Oct4A protein was localized to the nucleus, whereas Oct4B and Oct4B1 were predominantly expressed in the cytoplasm (Wang and Dai, 2010). Among the three isoforms, the function of Oct4A is the best known and widely described as a transcription factor responsible for pluripotency and self-renewal of the embryonic stem cells (ESCs) (Wang and Dai, 2010). In addition, Oct4A has been proven to be closely related to CSC-like properties, even as a CSCs marker in liver cancer (Wang X.Q. et al., 2010; Murakami et al., 2015; Liu et al., 2017) and in some other malignancies (Cho et al., 2018). Recent studies have reported that Oct4A is also relevant to angiogenesis. Direct endothelial lineage reprogramming can initiate through the introduction of Oct4 and other transcription factors. In these cases, Oct4 has been reported as a master regulator for the endothelial lineage transition from human iPSCs (Margariti et al., 2012; Li et al., 2013; Yoo et al., 2013). In addition, Hess et al. (2019) demonstrated that Oct4 plays an essential role within the perivascular cells in injury- and hypoxia-induced angiogenesis. All these reports together indicate that Oct4 participates in angiogenesis as a master regulator in normal stem cells. Furthermore, Oct4A^+^ cells were proven to act as progenitors of TECs in tumor (Pezzolo et al., 2011). As for other isoforms, the exact biological functions remain unclear for Oct4. Some studies have reported the potential contributions of Oct4B1 and Oct4B to the malignant properties of tumor cells, including invasion, anti-apoptotic, and chemotherapy resistance (Wen et al., 2015; Lin et al., 2019). However, the involvement of Oct4 isoforms in the vasculogenic potential of CSCs remains to be fully elucidated.

Here, we set out to investigate the roles of Oct4 in promoting vasculogenesis of LCSCs. We hypothesized that Oct4 may be regulate the endothelial transition of LCSCs. We previously discovered that a single cell-cloned LCSC, T3A-A3, has the potential to transdifferentiate into various tumor cells under given conditions and that Oct4A plays the pivotal role in these events (Liu et al., 2013). Thus, we believe that the present study will offer evidence to detect the effect of Oct4 on vasculogenesis in LCSCs. We searched for the isoforms of Oct4 involved in this process, and demonstrated that LCSCs possess the *trans*-differentiation potential toward TECs but also hints about the differential role of Oct4A and Oct4B1 in the respective regulatory function of *trans*-differentiation and lineage-restricted differentiation for the endothelial lineage in LCSCs. This is the first study to reveal the potential involvement of two Oct4 isoforms (Oct4A and Oct4B1) in regulating the endothelial transition of LCSCs. We believe that our study will provide insights to the development of novel therapeutic strategies for the treatment of liver cancer, particularly with respect to anti-angiogenesis resistance.

## Materials and Methods

### Cancer Stem Cell Line and Reagents

We identified a single cell-cloned cancer stem cell, T3A-A3, derived from a hepatoma patient in our previous study (Liu et al., 2013).

AKT-activator SC79 [HY-18749, MedChemExpress (MCE)] was dissolved in dimethyl sulfoxide (DMSO), diluted with Gibco Dulbecco’s Modified Eagle Medium: Nutrient Mixture F-12 (DMEM-F12) medium to achieve 25-μM concentration, and finally cultured for 30 min for *in vitro* experiments. The treated cells were analyzed by immunoblotting, as per the standard protocols.

### Spheroids Culture and Endothelial *Trans*-Differentiation

CSCs were maintained in stem cell medium (SCM) for spheroids culture. The details can be seen in our previous research (Liu et al., 2013, #29). For endothelial differentiation, the spheroids of CSCs were dispersed to single cells and grown in endothelial growth medium (EGM) containing endothelial cell basal medium and endothelial cell growth supplements (EBM-2/EGM^TM–2^; LONZA, Walkersville, United States) for 14 days. All cells were maintained in a humidified chamber at 37°C under 5% CO_2_ atmosphere.

### Human Hepatocellular Carcinoma (HCC) Specimens

The human HCC specimens were collected by the China–Japan Friendship Hospital, China with informed consents from the patients or their guardians and with due approval from the Institutional Ethics Committee of China–Japan Friendship Hospital (#2017-27). Pathological diagnoses were confirmed with reference to the 4^th^ edition of the World Health Organization (WHO) Classification of Tumors of the Digestive System (Flejou, 2011).

### Tube Formation Assay

*In vitro* vasculogenic potential of LCSCs was examined by using the Angiogenesis Assay Kit (ECM625; Millipore, Billerica, MA, United States), as per the manufacturer’s guidelines. The details can be seen in our previous research (Kuang et al., 2016, #58).

### Flow Cytometry Assay

For the flow cytometry assay, the cells were blocked with 5% FBS for 15 min and incubated with primary and secondary antibodies or stained with direct fluorescent-labeled antibodies. The expression of markers CD105, CD144, VCAM-1, CD31, CD133, and Oct4 (BD Biosciences, San Jose, CA, United States) were analyzed by a flow cytometer (Canton Flow Cytometer, BD) as per the standard method.

### IHC, Dual Staining, and IF

IHC, Dual Staining, and IF analysis of xenograft tumors in NOD-SCID mice was conducted as per the standard protocols. Briefly, the tissue specimens were fixed in 10% formalin, embedded in paraffin, sectioned, deparaffinized, and subjected to microwave antigen retrieval, followed by the inhibition of the endogenous peroxidase activity. The sections were subsequently stained. The details can be seen in our previous papers (Liu et al., 2013, #29; Kuang et al., 2016, #58).

### Confocal Microscopy

Confocal microscopy was performed as suggested elsewhere (Cheng et al., 2018). Briefly, the cells were fixed in 4% paraformaldehyde at the room temperature for 10 min. Then, the cells were blocked with 0.5% BSA for 30 min at the room temperature, followed by incubation with the antibodies CD31 (1:100; #3528; Cell Signaling) and Oct4 (1:200; #2750; Cell Signaling) for overnight at 4°C. Finally, incubated with anti-mouse and anti-rabbit IgG, respectively, conjugated with Alexa Flour 488 (1:1000; A-10680; Thermo) and Alexa Flour 647 (1:1000; A-32795; Thermo) for 50 min at the room temperature. Subsequently, the coverslips were treated with DAPI (P36962; Thermo) and visualized under a laser scanning confocal microscope (A1+; Nikon).

### RNA Isolation and Real-Time PCR Analysis

RNA was extracted by using the RNeasy mini kit (#74134, Qiagen, United States) following the instructions of the manufacturer. One microgram RNA was reverse-transcribed by Reverse Transcription System (RR047A, Takara, Japan). The expression of Oct4 was detected by quantinova Q5 Real-time PCR System (Thermo) using the QuantiNova SYBR Green PCR Kit (#208054, Qiagen) according to the manufacturer’s instructions. The specific primers for Real-time PCR were the following:

βNA was e

forward, 5′-TGGCACCACACCTTCTACAATGAGC-3′,

reverse, 5′-GCACAGCTTCTCCTTAATGTCACGC-3′;

Oct4A:

forward, 5′-TCGCAAGCCCTCATTTC-3′,

reverse, 5′-CATCACCTCCACCACCT-3′.

### Western Blotting

Western blotting (WB) was carried out routinely. The primary antibodies used here included anti-Oct4 (1:1000, #2750; Cell Signaling), CD144 (1:1000, #2500; Cell Signaling), CD105 (1:1000, ab11414; Abcam), CD31 (1:1000, #3528; Cell Signaling), mouse anti-human GAPDH (1:10000, HRP-60004; proteintech) and mouse anti-human β-actin monoclonal antibody (1:10000, A5316; Sigma). Protein bands were visualized with SuperSignal^TM^ West Pico PLUS Chemiluminescent Substrate (#34580; Thermo).

### Knockdown of Oct4A by Lentivirus-Mediated shRNA

T3A-A3 cells were infected with pLVTHM-shOct4 lentivirus vector or a control vector pLVTHM-scramble (kindly provided by Prof. Kosik). The Oct4A-targeting shRNA sequence (5′-AACATGTGTAAGCTGCGGCCC-3′) and the non-silencing control sequence (5′-GCTTGTTCGTTGGTAACTACA-3′) have been validated previously in other team (Xu et al., 2009) and our previous research. The details can be seen in our previous papers (Liu et al., 2013, #29).

### *In vivo* Xenograft Tumor

All animal studies were approved by the Animal Ethics Committee of China-Japan Friendship Hospital and were conducted in accordance with the Guide for the Care and Use of Laboratory Animals. Four-week-old NOD-SCID mice were provided by Charles River Laboratories (Beijing, China). These mice were apportioned into 2 groups of 5 each that were subcutaneously injected with 5 × 10^5^ shOct4-T3A-A3 cells and shNC-T3A-A3 cells, respectively. At the end of 4^th^ week of the administration, tumors were removed and sampled for the IF and IHC assays.

### Suppression of Oct4B1 by siRNAs

Two siRNAs specific to Oct4B1 and an irrelevant siRNA (used as a negative control) with the following sense and antisense sequences were used in this experiment: Oct4B1 siRNA No. 1, 5′-GGAGUAUCCCUGAACCUAGTT-3′ (sense) and 5′-CUAGGUUCAGGGAUACUCCTT-3′ (antisense); Oct4B1 siRNA No. 2, 5′-GAGGUGGUAAGCUUGGAUCTT-3′ (sense) and 5′-GAUCCAAGCUUACCACCUCTT-3′ (antisense); and the control siRNA, 5′-UUCUCCGAACGUGUCACGUTT-3′ (sense) and 5′-ACGUGACACGUUCGGAGAATT-3′ (antisense). All siRNAs were designed using primer 5.0 and synthesized by the GenePharma Corporation (Suzhou, China). T3A-A3 cells were transfected with 50 nmol/mL Oct4B1-1-siRNA (siOct4B1-1), OCt4B1-2-siRNA (siOct4B1-2), or siNC. Lipofectamine RNAIMAX (#13778075; Thermo) and Opti-MEM media (#31985062; Thermo) were used for the transfection process. After 48 h of transfection, the cells were used for further experiments.

### Statistical Analysis

All data were obtained from multi-replicated experiments and presented as the mean ± standard deviation (SD) or in other forms. Mann–Whitney non-parametric test and unpaired or paired Student’s *t*-test were selected to compare the mean values in two group comparisons. Linear regression model was applied to test the correlation extent between the two quantitative variables. All statistics were conducted by using the GraphPad Prism 8.0 software. Statistical significance was assigned at *P* < 0.05.

## Results

### Oct4 Is Closely Related to Tumor Vasculogenesis in Human HCC Specimens

**FIGURE 1:**
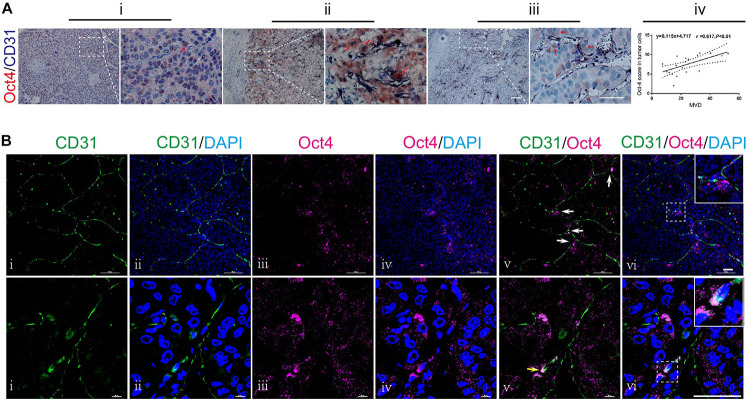
Anatomical relationship between Oct4 and vascular endothelial cells in human HCC. **(A)** Double immuno-histochemical staining for Oct4 (red) and CD31 (dark-blue). Stronger nuclear staining of Oct4 in poor-differentiated tumors (i) and more cytoplasmic distribution of Oct4 in well-differentiated ones (ii). Oct4^+^ tumor cells closely located to CD31^+^ vessels (ii, iii). The right panels show partial enlargements of dotted dashed-line boxes from the corresponding left panels. Red arrows: Oct4 staining. Blue arrows: CD31 staining. Scale bar: 100 μm. A statistical trend toward a positive correlation between the Oct4 expression and MVD (iv). MVD, microvessel density. **(B)** Confocal laser scanning imaging for Oct4 (purple) and CD31 (green). White arrows: Oct4^+^ tumor cells next to the branch point of CD31^+^ vessel (upper panel). Yellow arrow: co-expression of CD31 and Oct4 in endothelial cells on the luminal surface of vascular endothelium (lower panel). The insets display the enlarged views from dashed-line boxes. Nuclei are stained with DAPI (blue). Scale bar: 50 μm.

To investigate the relationship between Oct4 and vasculogenesis in hepatoma, we examined 20 HCC specimens by performing Oct4 and CD31 double-labeled immunohistochemistry (IHC) assay. We detected Oct4 both in the nuclei (Figure 1A-i; red arrow) and the cytoplasm of tumor cells (Figure 1A-ii; red arrows). Notably, we observed that some of the Oct4-positive neoplastic cells were located close to the CD31-positive vessels (Figure 1A-ii/iii, blue arrows). Furthermore, statistical analysis revealed a trend of positive correlation between the Oct4 expression and the microvessel density (MVD) (Figure1A-iv).

A more meticulous observation was performed by confocal laser scanning microscopy. The tumor vessels were marked by CD31-immunostaining. We found that some of the Oct4^+^ neoplastic cells were distributed along the orientation of CD31^+^ vessels, while focally aggregating at vessel branch points (Figure 1B; upper panel). We further examined the immunophenotypic changes in neoplastic cells adhering to vessel sprouts and captured the overlapping images of CD31 and Oct4 in the same cells (Figure 1B; lower panel, yellow arrow), which demonstrated the co-expression of CD31 and Oct4 in endothelial cells on the luminal surface of vascular endothelium.

These findings indicate a close relationship between the Oct4 expression and vasculogenesis of hepatoma by tumor cells, especially by perivascular tumor cells. Furthermore, T3A-A3 LCSCs were used to detect the endothelium transition in order to ascertain the effect of Oct4 on vasculogenesis in hepatoma.

### Detection of Significantly Upregulated Expressions of Endothelial Markers in LCSCs Under *in vitro* Induction With EGM

To detect the capacity of LCSCs to transdifferentiate into endothelial-like cells *in vitro*, single cell-cloned T3A-A3 cells were cultured in EGM for 2 weeks. T3A-A3 cells cultured in SCM served as the control. Next, endothelial markers were detected at the protein levels by western blotting and immunofluorescence (IF) assay.

**FIGURE 2:**
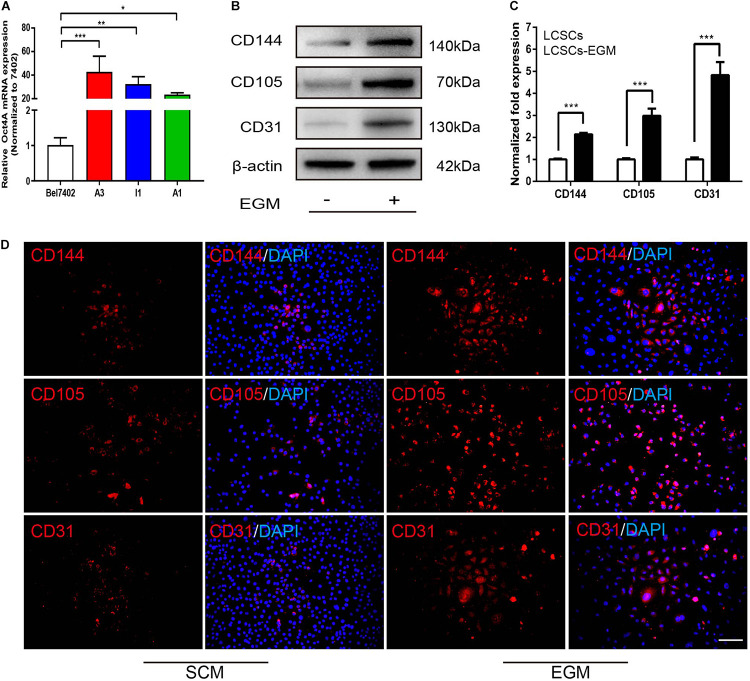
Differential expression of endothelial markers in SCM/EGM-cultured LCSCs *in vitro*. **(A)** Real-time PCR detected the expressions of Oct4 in different types of cells. **(B)** Comparison of the expressions of endothelial markers in LCSCs with or without EGM (SCM) induction by western blotting. **(C)** Quantitative analysis of the protein expression levels assessed by western blotting. **(D)** IF staining revealed that the expressions of endothelial markers were increased in LCSCs cultured with EGM compared to that of cultured with SCM. Nuclei are stained with DAPI (blue). *P < 0.05; **P < 0.01; ***P < 0.001. Scale bar: 100 μm. EGM, endothelial growth medium.

Real-time PCR revealed that Oct4A was highly expressed in T3A-A3 cells compared with some of other T3A clones and hepatoma cell line Bel7402 (Figure 2A), while CD144, CD105, and CD31 were markedly expressed in EGM-induced T3A-A3 cells than in control cells (Figure 2B). Quantitative analysis of the western blotting results confirmed the significant upregulation of the three proteins in EGM-induced T3A-A3 cells (Figure 2C). Moreover, similar changes were recorded by IF assay (Figure 2D). Cumulatively, these data provided the preliminary evidence for the *in vitro* endothelial *trans*-differentiation potential of LCSCs under specific stimulus condition.

### LCSCs Exhibit Vasculogenic Activity Under *in vitro* EGM Induction

**FIGURE 3:**
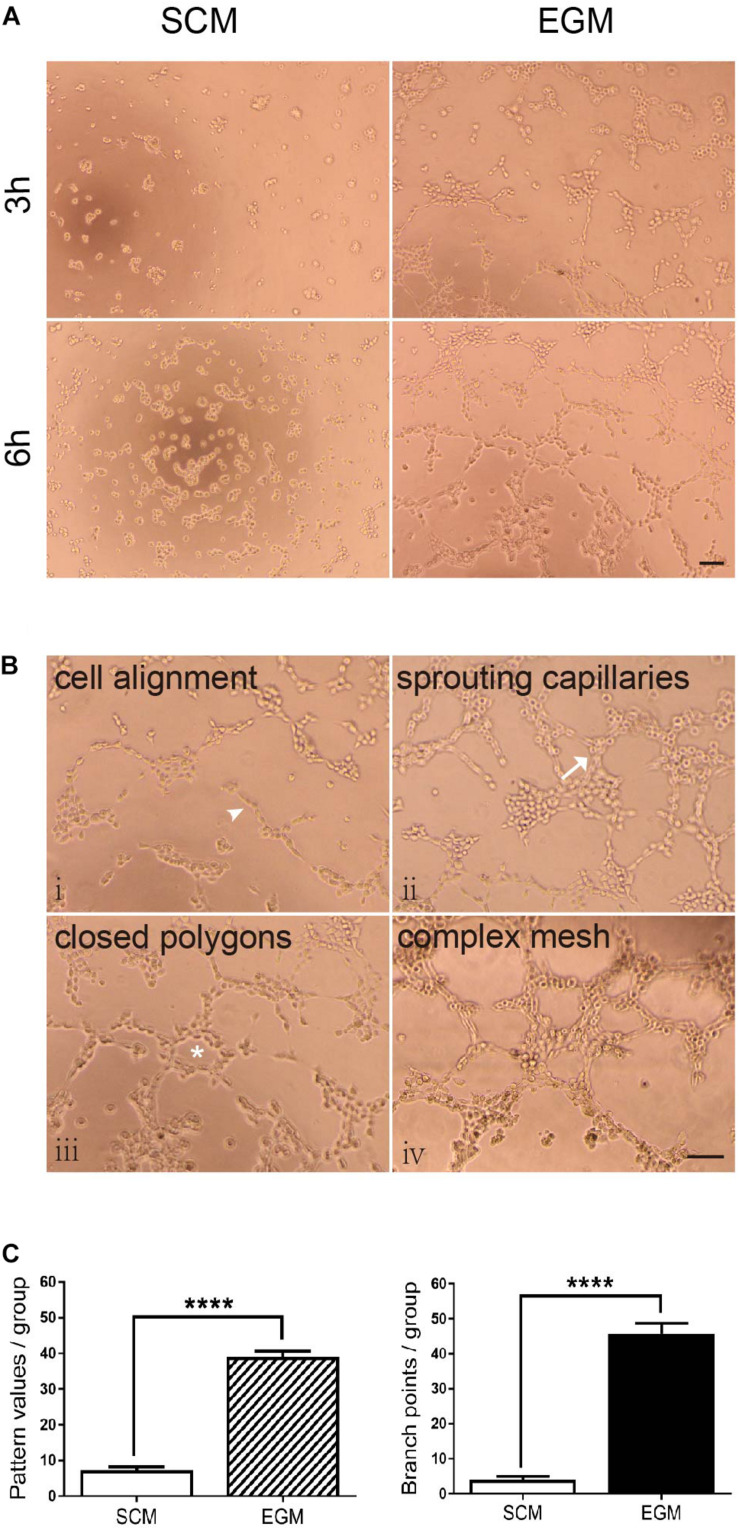
Increased tubulogenic capacity of LCSCs under *in vitro* EGM induction. **(A)** No tube formation was observed in LCSCs cultured with SCM medium (left panel); however, tube formation could be observed at the 3rd hour of the experiment, and it fully developed to a complex mesh-like structure at the 6^th^ hour in EGM-induced LCSCs (right panel). **(B)** Pattern recognition for tubulogenesis of LCSCs induced with EGM (i–iv). Arrowhead: cell alignment. Arrow: sprouting capillaries. Star: closed polygon. **(C)** Quantified analysis of tubulogenic capacity in each group according to the “pattern recognition” (left) and “branch point counting” (right). ^****^P < 0.0001. Scale bar: 100 μm.

*In vitro* tubulogenesis assay was performed for functionally testing whether LCSCs possess the vasculogenic activity after EGM induction. The tube formation in each group was monitored in real-time by phase-contrast microscope. Control T3A-A3 cells did not show any tubule formation; the cells were either scattered or randomly migrating. In contrast, EGM-induced T3A-A3 cells began to form tubules at the 3rd hour of the experiment. Subsequently, tube formation by these cells was fully developed by 6 h of the experiment (Figure 3A). The initiation and development of tubulogenesis were observed and identified, along with events such as cell alignment, sprouting of capillary tubes, and the formation of closed polygons and complex mesh-like structures (Figure 3B, i–iv).

The tubulogenic capacity of each group was quantified as per two evaluation criteria: “pattern recognition” and “branch point counting” (Figure 3C). Statistical analysis revealed that tubulogenesis of LCSCs significantly increased under EGM induction as compared with that under control condition (*P* < 0.0001). These findings suggest that the *in vitro* vasculogenic potential of LCSCs could be conditionally activated.

### LCSCs Give Rise to Vascular Endothelial Cells Participating in Tumor Vessel Formation in Xenografted Tumors

**FIGURE 4:**
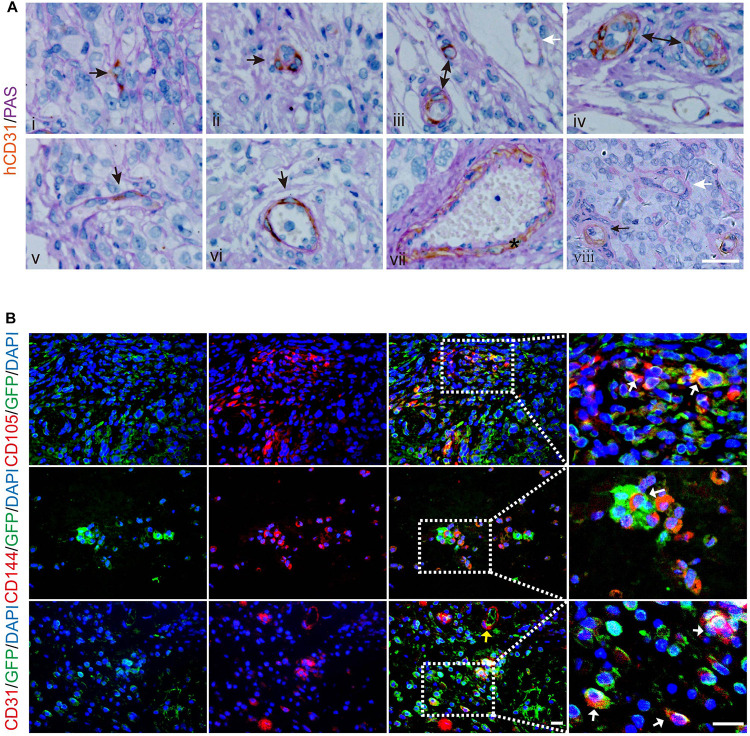
Incorporation of human LCSCs into vasculature of xenografted tumors. **(A)** Double histochemical staining of hCD31 (brown) and PAS (purple-red) in the xenografts. Sequential development of microvessels constituted by hCD31^+^ cells (i–iv). The hCD31^+^ cells incorporated into the endothelium of tumor vessels at different ratios (v,vi). Black arrows indicating hCD31 staining. The star denotes a larger hCD31^+^ vessel (vii). White arrows indicate vessel negative for hCD31 (iii, viii). **(B)** IF staining (red) for human-specific CD105 (upper panel), CD144 (middle panel), and CD31 (lower panel) in tumors derived from LCSCs labeled with GFP (green). Right panels are partially enlarged views from dotted dashed-line boxes. Yellow arrow: hCD31^+^ microvessel. White arrows: cells overlapping GFP and hCD31, hCD105, or hCD144. Nuclei are stained with DAPI (blue). Scale bar: 50 μm. GFP, green fluorescent protein.

Next, we attempted to validate the capacity of LCSCs for differentiating into tumor endothelial-loke cells *in vivo*. For this purpose, T3A-A3 cells were subcutaneously injected into 4-week-old non-obese diabetic/severe combined immunodeficiency (NOD-SCID) mice. We examined the T3A-A3 xenografts by dual histochemical staining for the pan-endothelial marker CD31 and PAS. Human-specific CD31 (hCD31) expression was detected in some anaplastic single cells and cell clusters (Figure 4A-i/ii). Moreover, such human CD31-positive cell clusters, which are enclosed by PAS-reactive basement membrane-like matrix, form microvessel-like structures and microvessels (Figure 4A-iii/iv). Among these tumor vessels, a few vessels contained a small amount of hCD31^+^ TECs (Figure 4A-v), while most of the vascular endothelia were constituted by hCD31^+^ TECs (Figure 4A-vi). In addition, some larger vessels were also strongly immunostained with hCD31 (Figure 4A-vii). Moreover, we also observed that the hCD31^+^ vessels were mingled with hCD31- vessels (Figure 4A-iii/viii), of which the latter could have been host-derived vessels. We next determined the participation of LCSCs in tumor vessel formation through immunobiological analysis.

To further confirm that the tumor endothelial-loke cells are derived from transplanted LCSCs, we applied green fluorescent protein (GFP)-labeling technique to track the T3A-A3 cells. The tissue sections of the xenografted tumors were immunostained for human-specific CD105, CD144, and CD31 by IF. We detected CD105, hCD144, and hCD31 proteins in some of the GFP^+^ cells (Figure 4B; upper/middle panel), which indicated that the transplanted LCSCs had differentiated toward tumor endothelial-loke cells. In addition, the hCD31^+^ microvessels co-expressed with GFP (Figure 4B; lower panel, yellow arrow), which implied that transplanted LCSCs participated in the formation of blood vessels in xenografts. We verified these observations in 6/6 xenografts, which altogether suggested that the contribution of LCSCs to neovasculogenesis could not have been an accidental event during the growth of grafted tumors.

### Knockdown of Oct4A Results in Decreased Endothelial *Trans-*Differentiation Potential of LCSCs Under Endothelial Induction

**FIGURE 5:**
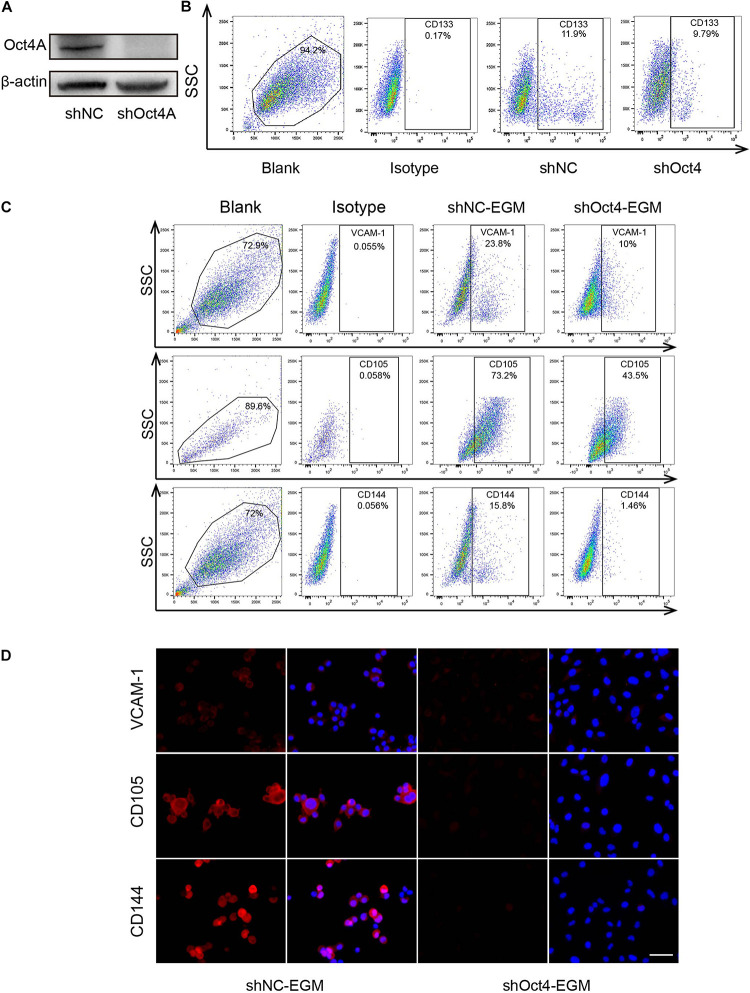
Knockdown of Oct4A inhibits the endothelial *trans*-differentiation potential of LCSCs under endothelial conditions. **(A)** Western blotting detected the efficiency of Oct4A knockdown. **(B)** Flow cytometry identified the corresponding decline in the CD133 expression. **(C)** Flow cytometry verified decrease in the specific endothelial markers of shOct4A-T3A-A3 as compared with that of shNC-T3A-A3 under the same endothelial conditions. **(D)** IF assay obtained the same results. Nuclei are stained with DAPI (blue). Scale bar: 100 μm.

To detect the effect of Oct4 on the *trans*-differentiation potential of CSCs in hepatoma, we conducted a shRNA - mediated knockdown of the Oct4A in T3A-A3 LCSCs and performed western blotting to confirm the decreased expression of Oct4A (Figure 5A). Simultaneously, we detected that the CD133 expression, which is another key LCSCs marker, also reduced by the fluorescent-activated cell sorting (FACS) method (Figure 5B), implying that the knockdown inhibits the stemness of CSCs. Next, we detected the obvious decline in the expression of endothelial markers of shOct4-T3A-A3 by FACS in comparison with that in shNC-T3A-A3 under EGM induction (Figure 5C). The same results were obtained by the IF assay and hence verified a few endothelial markers reduced in shOct4-LCSCs (Figure 5D). These results suggest that LCSCs lost their endothelial *trans*-differentiation potential *in vitro* after the Oct4 knockdown.

### ShOct4A-LCSCs Exhibit Endothelial-Like Properties Without Endothelial Induction

**FIGURE 6:**
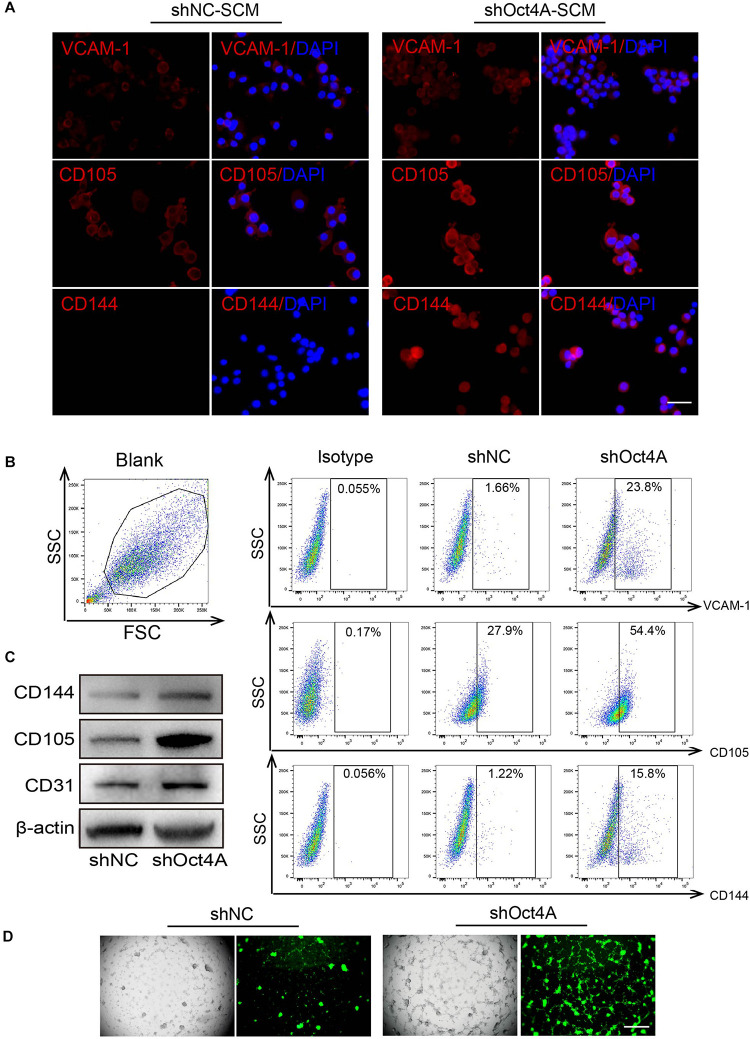
ShOct4A-LCSCs exhibit endothelial-like properties without endothelial induction. **(A)** IF detected the expressions of increasing endothelial markers in LCSCs after the knockdown of Oct4A under the non-endothelial (SCM) condition. **(B)** A similar trend was also observed by FACS. **(C)** WB identified the same results. **(D)** Vascular-like structure could be observed in shOct4A-LCSCs under non-endothelial condition by Angiogenesis Assay. Scale bar: 100 μm.

Surprisingly, we detected that the expression of endothelial markers increased in shOct4A-T3A-A3, such as that of VCAM-1, CD105, and CD144 in comparison with that in shNC by IF (Figure 6A) when cultured in the SCM but not in the EGM conditions. A similar trend was also observed by FACS (Figure 6B). In addition, the difference in the endothelial expression between shNC and shOct4A groups were further verified by western blotting (Figure 6C). Finally, during the investigation of *in vitro* vasculogenic potential, we detected vascular-like structure in shOct4A-T3A-A3 cells, but not in shNC cells, though under SCM condition (Figure 6D). All these results cumulatively implied that knockdown of Oct4A induces endothelial lineage-restricted differentiation of LCSCs.

### Knockdown of Oct4A Promotes the Nucleocytoplasmic Dynamics of Oct4 Transformation in LCSCs

**FIGURE 7:**
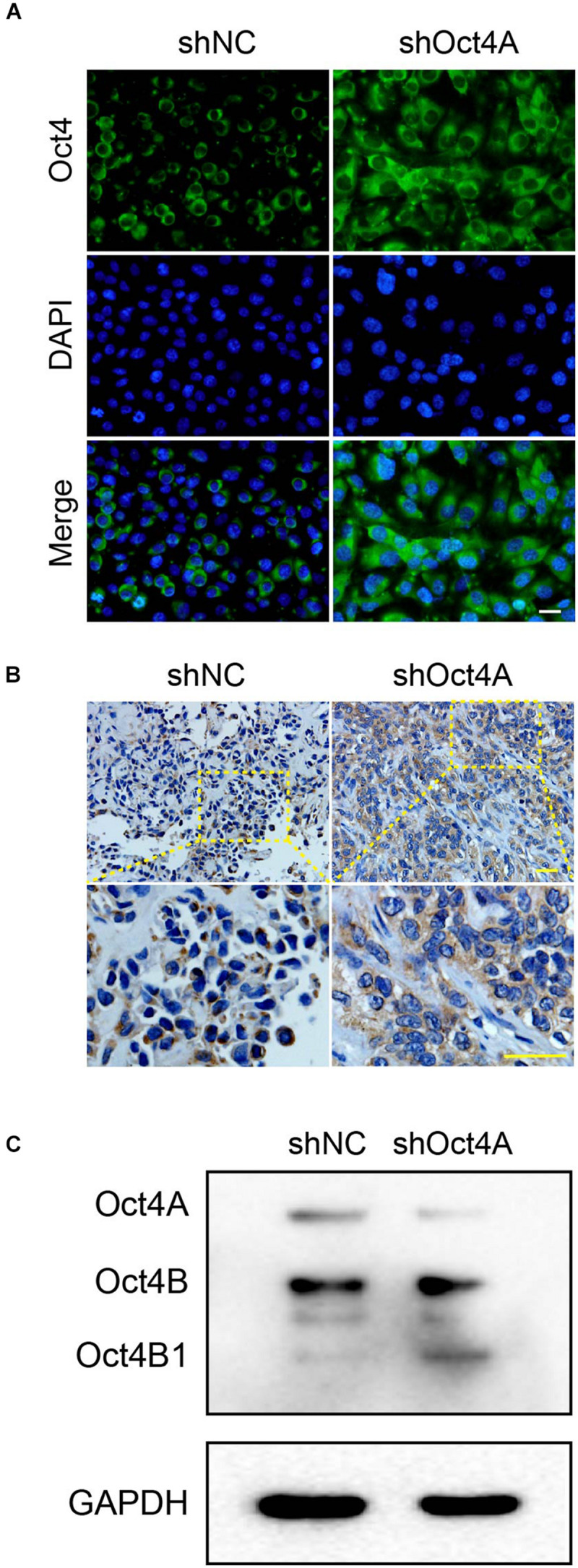
Changes in subcellular distribution of Oct4 after the knockdown of Oct4A in LCSCs. **(A)** IF assay detected cell localization of Oct4, mainly in the cytoplasm, in shOct4A-LCSCs, albeit shNC-LCSCs were located mainly in the nuclei *in vitro*. **(B)** IHC assay obtained similar results for xenograft tumor. **(C)** WB detected various isoforms of Oct4 and further verified the difference in their expression. Scale bar: 50 μm.

To further detect the role of Oct4 in endothelial lineage-restricted differentiation of LCSCs, we examined the subcellular distribution of Oct4. For this purpose, we used an antibody for Oct4 that could identify the total proteins of Oct4, including its three isoforms. Unexpectedly, the cell localization of Oct4 occurred mainly in the nuclei in shNC-LCSCs has changed into the cytoplasm of Oct4 in shOct4A-LCSCs *in vitro* by IF (Figure 7A) and in xenograft by IHC assay (Figure 7B). Both the morphological experiments implied the occurrence of nucleocytoplasmic translocation of the Oct4 expression after the knockdown of Oct4A.

In addition, to identify the various isoforms of Oct4, we performed the western blotting assay and confirmed three distinct bands of approximate molecular weights 45, 30, and 18 kDa, which corresponded to Oct4A, Oct4B, and Oct4B1, respectively. We also verified that the expression of only Oct4B1 increased among the three isoforms, while that of the other two declined (Figure 7C). All these results indicate that the knockdown of Oct4A promotes the expression of cytoplasmic Oct4 in LCSCs and hints that Oct4B1 may be a potential regulator of endothelial lineage-restricted differentiation.

### Interference of Oct4B1 Inhibits the Lineage-Restricted Differentiation of LCSCs Into Endothelial-Like Cells

**FIGURE 8:**
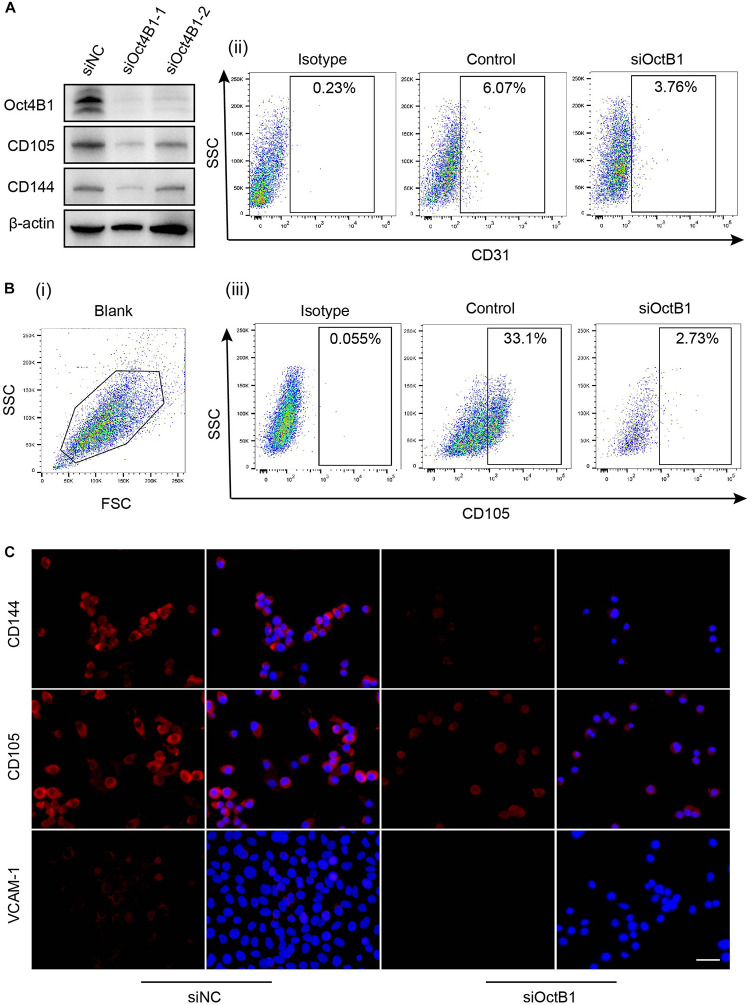
Interference of Oct4B1 regresses the expressions of endothelial markers in LCSCs. **(A)** WB identified the efficiency of knockdown of Oct4B1 and the decline in the expressions of endothelial markers, especially in siOct4B1-1. **(B)** FACS verified the decline of endothelial marker expression in siOct4B1-1-LCSCs. **(C)** IF obtained similar results that the expressions of endothelial markers sharply decreased in siOct4B1-1- LCSCs than in siNC- LCSCs. Scale bar: 100 μm.

To further verify whether Oct4B1 is involved in the regulation of the endothelial phenotype of LCSCs, we specifically inhibited the Oct4B1 expression with two pairs of siRNAs. We observed the following change by WB: two pairs of primers of siRNA-Oct4B1 successfully inhibited the expression of Oct4B1 in LCSCs, especially the siRNA1, and the downregulation of Oct4B1 remarkably decreased the expressions of human endothelial markers such as CD105 and CD144 (Figure 8A). Next, we used siOct4B1-1 to inhibit the Oct4B1 expression. The expression of CD31, CD105, and VCAM-1 were estimated by FACS (Figure 8B) and IF (Figure 8C). Similar results by both the tests confirmed that the expression of endothelial markers decreased in siOct4B1-1-LCSCs as compared to that in siNC. All these results indicate that Oct4B1 may be an indispensable factor for maintaining the endothelial phenotype in hepatoma CSCs.

### Oct4 Regulates Endothelial Lineage-Restricted Differentiation May Be Through the AKT/NF-κB-p65 Pathway Activation

**FIGURE 9:**
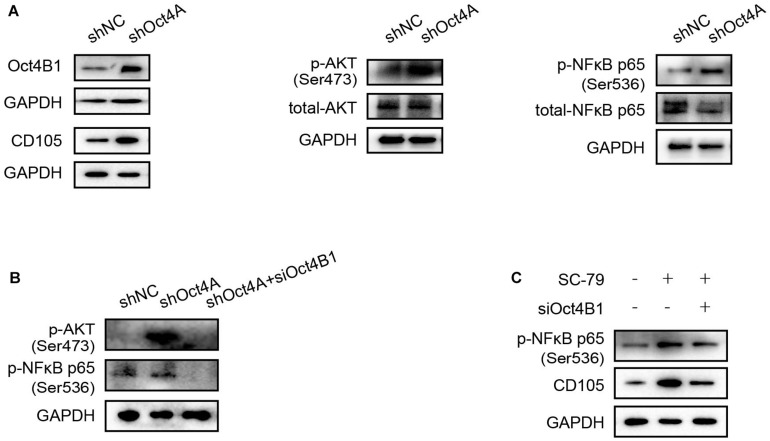
Oct4 regulates endothelial lineage-restricted differentiation through the AKT/NF-κB-p65 pathway. **(A)** WB detected that the knockdown of Oct4A activated p-AKT, p-p65, and CD105; these changes are in line with the expression of Oct4B1. **(B)** siOct4B1 can reverse the expression trend of AKT/NF-κB-p65 by the knockdown of Oct4A. **(C)** siOct4B1 can reverse the expression trend of NF-κB-p65, CD105 by AKT activator SC-79.

AKT and NF-κB-p65 are important signaling pathways in angiogenesis (Huang et al., 2018; Si et al., 2018). In addition, several studies have confirmed that Oct4 can induce the AKT/NF-κB-p65 activation (Campbell and Rudnicki, 2013; Li et al., 2017). In our study, When the Oct4A was knocked down by shRNA, the expression of Oct4B1 increased as also of the CD105. At the same time, AKT and NF-κB-p65 were activated (Figure 9A). When siOct4B1 was further utilized in the former cells, the expression of p-AKT and p-NF-κB-p65 were reversed (Figure 9B). We hence speculated that the interference of Oct4B1 downregulates the endothelial markers through the AKT/NF-κB-p65 pathway. Then, SC-79, an activator of AKT, was used to further confirm this speculation. The elevation of p-NF-κB-p65 by SC-79 was attenuated after interfering with Oct4B1 (Figure 9C). These results cumulatively suggest that Oct4B1 may accumulate CD105 through the activation of the AKT/NF-κB-p65 pathway.

## Discussion

In this study, we found that LCSCs could transit into tumor endothelial-like cells and participate in the vasculogenesis of hepatoma. The identification of the regulation of Oct4 in the endothelial transition of LCSCs confirms the involvement of this protein in the vasculogenesis of hepatoma through endothelial *trans*-differentiation, which is regulated via Oct4A, and through endothelial lineage-restricted differentiation, which is mediated by Oct4B1.

Since Folkman proposed the anti-angiogenic therapy of tumor in 1971, a large number of studies have focused on the mechanisms of tumor vessel formation. Recent studies have paid attention to the generation of TECs, among which the endothelial *trans*-differentiation of CSCs has been demonstrated in glioblastoma and other malignancies (Tang et al., 2016; Shangguan et al., 2017; Cui et al., 2018). Several different regulatory factors, including Notch-1 (Cui et al., 2018) and CCL5 (Tang et al., 2016), are reportedly involved in the molecular mechanism of tumor-endothelial *trans*-differentiation. However, whether CSCs can give rise to TECs in other cancer types remains to be confirmed, for which the molecular mechanism should be clarified.

We have been extensively pursuing the exploration of pluripotency of LCSCs. During one of our study, we sorted and identified T3A-A3 cells, a single-cell cloning of human LCSCs (Liu et al., 2013). In the present study, endothelial *trans*-differentiation of LCSCs was induced *in vitro* by EGM, as also applied by Soda et al. (2011). On the other hand, the vasculogenic potential of LCSCs was verified *in vivo*. An endothelial progenitor marker CD144 (Wang R. et al., 2010), activated the expression of tumor endothelial cell marker CD105, and the pan-endothelial marker CD31 (Hu et al., 2018) was used in combination to highlight the endothelial-like phenotype of LCSCs. Correspondingly, we selected the human-specific CD31 marker for the complete identification of vascular endothelial cells originating from human LCSCs, including active immature and stable mature cells in xenografts. Moreover, the incorporation of LCSCs into neovessels was tracked by labeling them with GFP *in vivo*. Furthermore, *in vitro* tubulogenesis—a functional characteristic of vascular endothelial cells—was observed in LCSCs under endothelial induction. Thereupon, the endothelial *trans*-differentiation potential of LCSCs was assessed phenotypically and functionally to provide new evidences of tumor-endothelial *trans*-differentiation. Importantly, such pluripotency has been proven to depend on specific exogenous stimulants, which conforms to our previous study (Liu et al., 2013) and other reports that showed the effect of a microenvironmental “niche” on the multidirectional differentiation of plastic cells (Ye et al., 2014).

Some past studies demonstrated that Oct4 is indispensable for the endothelial lineage transition in several types of pluripotent cells (Margariti et al., 2012; Li et al., 2013; Yoo et al., 2013). Our previous study has proved it to be essential for the pluripotency of LCSCs (Liu et al., 2013). In this study, we investigated the roles of Oct4 in regulating the endothelial differentiation potential of LCSCs.

We knocked down the Oct4A with shRNA in T3A-A3 cells. As identified by our and other previous studies (Xu et al., 2009; Liu et al., 2013), this shRNA of Oct4A can weaken the pluripotency of ESCs or LCSCs. The expressions of the endothelial markers in shOct4A-T3A-A3 cells decreased compared with that of in the shNC-T3A-A3 cells under the endothelial induction. This result implies that Oct4A is a crucial factor in regulating the endothelial *trans*-differentiation of LCSCs. This result is consistent with that of a previous study that confirmed the responsibility of Oct4A for pluripotency (Liu et al., 2015).

On the contrary, increased the endothelial expressions were detected when the shOct4A-T3A-A3 cells were cultured in conventional medium SCM, without endothelial induction. This result indicated that the Oct4A knockdown promotes the endothelial lineage-restricted differentiation, which is consistent with that of previous study the implication of Oct4A for stemness.

Further study revealed that the cellular localization of Oct4 in LCSCs changed from the nuclei to the cytoplasm along with increased expression of the endothelial maker after the knock down of the Oct4A. This finding suggests that the nucleocytoplasmic translocation of Oct4 may be involved in the regulation of endothelial lineage-restricted differentiation in LCSCs. Oka et al. (2013) also proposed that Oct4 is a nucleocytoplasmic shuttling protein. Our study results thus favor the assumption by Oka’s report and together provide new insights to the role of Oct4 in regulating the endothelial lineage transition of CSCs.

Because of distinct subcellular distribution of Oct4 isoforms, we speculated that the increasing cytoplasmic Oct4 is either due to Oct4B or Oct4B1. We then selected an anti-Oct4 antibody that could recognize the endogenous total protein of Oct4 to perform the WB assay, and clearly captured the expression of Oct4 isoforms of different molecular weights: a single promotion at the 18-kDa position only was noted, which matched the molecular weight of Oct4B1. To further verify the above results, we knocked down Oct4B1 by using siRNAs, and the decreased endothelial markers were detected at the protein levels by WB, FACS, and IF methods.

**FIGURE 10:**
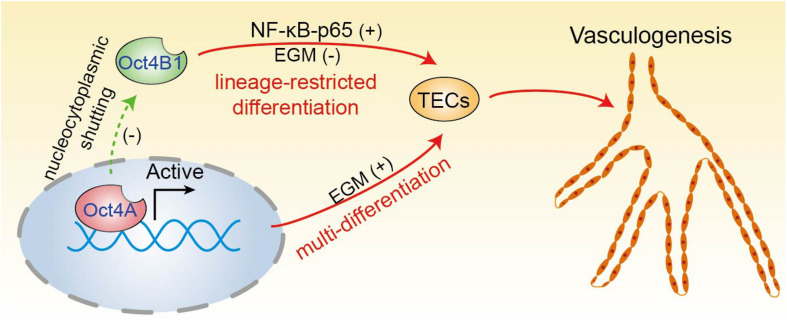
A proposed model of two Oct4 isoforms involved in the endothelium-oriented transition of LCSCs. Oct4 regulates the TECs transition of LCSCs by at least two means: LCSCs trans-differentiate into TECs regulated by Oct4A, which play a crucial role in the maintenance of pluripotency of LCSCs; on the other hand, LCSCs can lineage-restricted differentiate into TECs directly through the positive regulation of Oct4B1. In addition, Oct4A may negatively regulate Oct4B1 by nucleocytoplasmic shutting.

Altogether, all of our findings and well-known literature data depict a rough outline for the potential involvement of the two Oct4 isoforms in the endothelial lineage transition of LCSCs (Figure 10). The high expression of Oct4A (the nuclear isoform of Oct4) maintains the pluripotency of LCSCs and regulates LCSCs trans-differentiate into tumor endothelial-like cells. The downregulation of Oct4A is accompanied with the upregulation of Oct4B1—a cytoplasmic isoform of Oct4—which regulates the process of LCSCs undergoing a direct endothelium-oriented restricted differentiation, but not *trans*-differentiation regulated by Oct4A.

Moreover, we also examined the Oct4 expression pattern in human liver cancer specimens. We detected Oct4 proteins that were correspondingly expressed both in the nuclei and cytoplasm of liver cancer cells. However, we also noticed that the focal aggregation of some of the Oct4 cytoplasmic positive tumor cells were especially next to the vessel branch points. In addition, we identified a positive correlation trend between the MVD and Oct4 expression in hepatoma cells. Notably, we detected the co-expression of Oct4 and CD31 not only in perivascular tumor cells but also in vascular endothelial cells. These phenomena confirmed the involvement of Oct4 in the neovasculogenesis of perivascular niche (PVN) in liver cancer. However, our results need to be confirmed at a larger-scale by histological analysis of human liver cancer.

This study is the first to demonstrate that CSCs can participate in vasculogenesis by transdifferentiating and lineage-restricted differentiating into tumor endothelial-like cells simultaneously, for which two types of Oct4 isoforms are responsible: Oct4A and Oct4B1 may be the key sites of endothelial lineage transition. However, these conclusions are based on the responses of liver CSCs, which may not reflect the processes that occur in other types of CSCs. Thus, additional studies are needed to validate these conclusions in diverse types of CSCs. We also detected that the AKT-NF-κB-p65 signaling pathway may be responsible for the regulation of Oct4 in the transition of tumor endothelial-like cells from LCSCs. Large-scale studies with longer duration of follow-up are hence necessary to evaluate the additional mechanisms of regulation of TECs by Oct4. In the future, we plan to not only decipher these regulation mechanisms but also elucidate the Oct4-mediated signaling pathways in the endothelial lineage transition.

## Data Availability Statement

All datasets generated for this study are included in the article.

## Ethics Statement

The human HCC specimens were collected by the China-Japan Friendship Hospital, with informed consents from the patients or their guardians and approval from the Institutional Ethics Committee of the China-Japan Friendship Hospital. The animal studies were reviewed and approved by the Animal Ethics Committee of China-Japan Friendship Hospital.

## Author Contributions

H-LL, X-YK, and Q-SL designed the conceptual framework of the study and analyzed and interpreted the data. H-LL conducted the major experiments. H-lY, H-tT, T-TD, S-QX, QF, and Y-PX performed the experiments. ZW and LP provided advices and supports. H-LL wrote the drafts. Q-SL and X-YK revised the manuscript. All authors have read and approved the final manuscript for submission to the target journal.

## Conflict of Interest

The authors declare that the research was conducted in the absence of any commercial or financial relationships that could be construed as a potential conflict of interest. The handling editor declared a past collaboration with one of the authors Q-SL.
